# Caspase-1 Deficiency Modulates Adipogenesis through Atg7-Mediated Autophagy: An Inflammatory-Independent Mechanism

**DOI:** 10.3390/biom14040501

**Published:** 2024-04-20

**Authors:** Yumeng Wang, Gaojun Chen, Min Xu, Yewei Cui, Weijiong He, Hongxiang Zeng, Ting Zeng, Rui Cheng, Xi Li

**Affiliations:** Institute of Life Sciences, School of Basic Medicine, Chongqing Medical University, Chongqing 400016, China

**Keywords:** Caspase-1, adipogenesis, autophagy, Atg7

## Abstract

Obesity stands as a significant risk factor for type 2 diabetes, hyperlipidemia, and cardiovascular diseases, intertwining increased inflammation and decreased adipogenesis with metabolic disorders. Studies have highlighted the correlation between Caspase-1 and inflammation in obesity, elucidating its essential role in the biological functions of adipose tissue. However, the impact of Caspase-1 on adipogenesis and the underlying mechanisms remain largely elusive. In our study, we observed a positive correlation between Caspase-1 expression and obesity and its association with adipogenesis. In vivo experiments revealed that, under normal diet conditions, Caspase-1 deficiency improved glucose homeostasis, stimulated subcutaneous adipose tissue expansion, and enhanced adipogenesis. Furthermore, our findings indicate that Caspase-1 deficiency promotes the expression of autophagy-related proteins and inhibits autophagy with 3-MA or CQ blocked Caspase-1 deficiency-induced adipogenesis in vitro. Notably, Caspase-1 deficiency promotes adipogenesis via Atg7-mediated autophagy activation. In addition, Caspase-1 deficiency resisted against high-fat diet-induced obesity and glucose intolerance. Our study proposes the downregulation of Caspase-1 as a promising strategy for mitigating obesity and its associated metabolic disorders.

## 1. Introduction

Obesity is a critical global health issue, characterized by a steady increase in its prevalence [1,2]. Since 1980, the worldwide prevalence of overweight and obesity has doubled, affecting nearly a third of the global population [3]. This alarming trend underscores obesity as a significant risk factor for a spectrum of metabolic disorders, including hypertension, dyslipidemia, and diabetes. Its pervasive impact spans nations, with projections indicating a worsening scenario in the coming decade. Consequently, this trajectory is anticipated to escalate the toll on quality of life, disability, and mortality rates [4,5].

The primary cause of obesity stems from an imbalance between energy intake expenditure, accumulating excess energy in the form of triglycerides. This results in an excessive buildup of lipid and adipose tissue enlargement [6]. The proper remodeling adipose tissue hinges on a delicate equilibrium between de novo adipogenesis, where mesenchymal stem cells differentiate into new adipocytes, and the enlargement of existing adipocytes (hypertrophy) [7,8,9,10]. Adipocyte hypertrophy is often accompanied by detrimental factors such as inflammation and fibrosis, which can contribute to elevated blood lipid and blood glucose levels [10]. In contrast, a healthy expansion of adipose tissue through adipogenesis involves an increase in the number of adipocytes without significant changes in adipocyte size. This process serves as a protective mechanism against metabolic disorders associated with obesity [8,9]. Adipogenesis is governed by the complex interplay of adipocyte-specific transcription factors and proteins such as peroxisome proliferator-activated receptor gamma (PPARγ), CCAAT/enhancer binding protein alpha (C/EBPα), and fatty acid-binding protein (Fabp4) [11,12,13]. These genes serve as crucial markers for adipocyte differentiation and the progression of adipogenesis.

Studies have elucidated the pivotal role of various events, encompassing extracellular signaling, transcriptional cascades, and epigenetic modifications, in orchestrating the differentiation of adipocytes [14]. Autophagy emerges as a central intracellular degradation system, wherein organelles and proteins are enveloped in lysosomes for degradation, thereby regulating cellular homeostasis [15]. The existing research underscores the significance of autophagy as a fundamental biological process crucial for life maintenance, with notable implications in controlling lipid accumulation and adipogenesis [16]. Notably, the downregulation of Atg7 in 3T3-L1 adipocytes has been observed to impede lipid accumulation and reduce the expression of adipocyte differentiation factors [17]. In addition, experiments have demonstrated that attenuating Atg5 or inhibiting autophagy or lysosomal function pharmaceutically impairs adipogenesis [18].

Obesity manifests with heightened inflammation levels characterized by immune cell infiltration, notably macrophage infiltration, and the increased secretion of pro-inflammatory cytokines such as IL-1β and IL-6 within adipose tissue [19,20,21]. This inflammation environment not only hampers the normal proliferation and differentiation of adipocytes but also impairs the insulin signaling pathway, fostering insulin resistance concurrently [22]. In obesity-associated inflammation, inflammasomes, particularly the NLRP3 inflammasome, play a pivotal role [23]. Caspase-1, also known as an interleukin-1beta-converting enzyme, is a critical effector protein in this process, collaborating with NLRP3 and ASC [24,25,26]. Intriguingly, existing research indicates a dual role for Caspase-1 in obesity. While some studies suggest that Caspase-1 deficiency enhances insulin sensitivity and reduces adipose tissue mass compared to wild-type mice [27], contradictory reports propose that Caspase-1-deficient mice are more susceptible to high-fat diet (HFD)-induced obesity and increased inflammation through the CCL2/C-C chemokine receptor 2 (CCR2) axis in adipose tissue [25]. These findings underscore the intricate interplay between Caspase-1, lipid metabolism, and obesity. Furthermore, regarding its impact on adipogenesis, a study has established that Caspase-1 deficiency fosters adipocyte differentiation [26], although the underlying mechanism remains elusive.

In our investigation, we revealed that Caspase-1 deficiency promotes adipogenesis in *Casp-1*^−/−^ mice. Delving deeper into the mechanistic aspect, our in vitro experiments using primary adipocytes revealed that Caspase-1 deficiency increases the expression of Atg7 protein, thereby activating autophagy and subsequently enhancing adipocyte adipogenesis. These findings provide a novel therapeutic target for addressing obesity and aberrant adipose tissue metabolism.

## 2. Materials and Methods

### 2.1. Animals and Treatments

C57BL/6J mice were purchased from the Experimental Animal Center of Chongqing Medical University (Chongqing, China). *Casp-1*^−/−^ mice were acquired from Jackson Laboratory and were generated by crossing *Casp-1*^−/−^ with *Casp-1*^−/−^ mice on a C57BL/6J background. Male C57BL/6J WT and *Casp-1*^−/−^ mice were on a standard diet prior to the experiments, and then the mice were fed with a normal chow diet (ND) (Beijing KEAO XIELI FEED, Beijing, China) or a high-fat diet (HFD) (60% fat; Research Diets, New Brunswick, NJ, USA) for 12 weeks from the 8th week of study. All the experiments were performed under the approved guidelines of the institutional Animal Care and Use Committee of Chongqing medical university and followed the National Institutes of Health’s guidelines on the care and use of animals.

### 2.2. Glucose and Insulin Tolerance Tests

For the glucose tolerance test (GTT), the mice were fasted for 14 h (from 20:00 to 10:00). After that, the mice were intraperitoneally injected with glucose (2 mg/g glucose, 50% glucose solution). The glucose level was monitored at the following time points: 30 min, 60 min, 90 min, and 120 min. For the insulin tolerance test (ITT), the mice were fasted for four hours (from 10:00 to 14:00). After that, the mice were intraperitoneally injected with insulin (0.75 mU/g). The glucose level was monitored at the following time points: 30 min, 60 min, 90 min, and 120 min.

### 2.3. RNA Preparation and RT-qPCR

RNA was extracted from cultured cells or frozen tissue samples using TRIzol (Invitrogen, Waltham, MA, USA). For quantitative real-time PCR analysis, 1 μg total RNAs were reverse-transcribed by using a Revert Aid first strand cDNA synthesis kit (Thermo Scientific, Waltham, MA, USA). The cDNA was analyzed using the Power SYBR green PCR master mix (Applied Biosystems, Carlsbad, CA, USA) with the ABI Prism 7500 qPCR machine (Applied Biosystems). mRNA qPCR data were normalized to 18 s. The primers used for RT-qPCR in this research were synthesized by Sangon Biotech and are shown in Supplementary Table S1.

### 2.4. Western Blotting and Antibodies

Tissue homogenates or cell lysates were derived from lysis buffer containing 2% SDS and 50 mM Tris–HCl (PH 6.8). The lysates were then quantitated, and equal amounts of protein were subjected to SDS-PAGE, then transferred to nitrocellulose membranes (Bio-Rad, Hercules, CA, USA). The membranes were probed with primary antibodies against Hsp90, β-actin, Caspase-1, C/EBPα, PPARγ, Fabp4, Beclin-1, Atg5, Atg7, and LC3A/B. Antibodies against Hsp90, PPARγ, p62, Beclin-1, Atg5, Atg7, and LC3A/B were obtained from Cell Signaling Technology, antibodies against Caspase-1, Fabp4 and C/EBPα were obtained from Santa Cruz Biotechnology, and an antibody against β-actin was obtained from Proteintech.

### 2.5. Cell Culture and Induction of Differentiation

The original 3T3-L1 cells we used were provided by Professor Qiqun Tang (Fudan University, Shanghai, China). The 3T3-L1 cells were propagated and maintained in DMEM containing 10% calf serum (Gibco, Grand Island, USA). To induce differentiation, two days post-confluence cells (designated day 0) were induced to differentiate with DMEM containing 10% fetal bovine serum (Gibco), 1 μg/mL insulin, 1 μmol/L dexamethasone, and 0.5 mmol/L 3-isobutyl-1-methylxanthine until day 2. The cells were then fed DMEM supplemented with 10% fetal bovine serum and 1 μg/mL insulin for 2 days; then, the cells were incubated in DMEM with 10% fetal bovine serum.

### 2.6. Isolation of SVF and SVF Culture (Primary Adipocytes)

Stromal vascular fraction (SVF) cells from white fat tissue were prepared using a previously reported method, with a few modifications. Briefly, sub-WAT was dissected and washed with PBS, minced, and digested by 0.075% collagenase (Sigma, Livonia, MI, USA) at 37 °C for 25–30 min. The digested tissues were filtered through a 100 μm mesh filter. After centrifugation, mature adipocytes floating above on the supernatant, and cellular pellets involving the SVF cells were resuspended with an ammonium chloride lysis buffer to remove red blood cells. Both the SVF cells and adipocytes were washed with 0.5% calf serum in phosphate-buffered saline (PBS). The freshly isolated SVF cells were seeded and cultured in F12:DMEM = 1:1 containing 10% FBS (Gibco) and 0.5% penicillin/streptomycin (P/S) at 37 °C with 10% CO_2_. On confluence, the cells were induced to differentiate for 2 days with F12:DMEM = 1:1 containing 10% FBS, 5 μg/mL insulin (Roche), 1 μmol/L dexamethasone (Sigma), 0.5 mmol/L of 3-isobutyl-methylxanthine (Sigma), and 5 μmol/L rosiglitazone (Sigma). The induction medium was replaced with F12:DMEM = 1:1 containing 10% FBS and 5 μg/mL insulin for 2 days. Then, the cells were incubated in F12:DMEM = 1:1 with 10% FBS. Autophagy inhibitors 3-MA (0.5 mM, dissolved in DMSO; Sigma) or CQ (2 µg/mL, dissolved in DMSO; MCE) were added to the culture medium during adipocyte differentiation.

### 2.7. RNA Interference

Primary adipocytes were transfected with siRNA using Lipofectamine RNAi MAX (Invitrogen) according to the manufacturer’s instructions. The sequences used for successful Atg7 knockdown were synthesized by Sangon, GCCUGGCAUUUGAUAAAUGUATT.

### 2.8. Oil Red O Staining and Quantification

In vitro-differentiated adipocytes were fixed for 30 min in buffered paraformaldehyde and stained with oil red O for at least 60 min. The stained fat droplets in the cells were visualized by light microscopy and photographed. For quantification, intracellular ORO was extracted with 100% isopropanol and quantified by measuring the optical absorbance at 490 nm using a microplate reader (Thermo).

### 2.9. Hematoxylin and Eosin (H&E) Staining

Paraffin-embedded adipose tissues were cut into 5 µm thick sections, adhered onto glass slides, deparaffinized, and rehydrated with ethanol with a decreasing concentration gradient. Tissue-embedded slides were stained with hematoxylin and eosin (H&E) and observed under a light microscope.

### 2.10. Statistical Analysis

The data were processed statistically using GraphPad Prism 9 software, and the results are expressed as mean ± SEM. Comparisons between groups were made using unpaired two-tailed Student *t*-tests, where *p* < 0.05 was considered as statistically significant

## 3. Results

### 3.1. Caspase-1 Expression Is Correlated with Obesity and Adipogenesis

**Figure 1:**
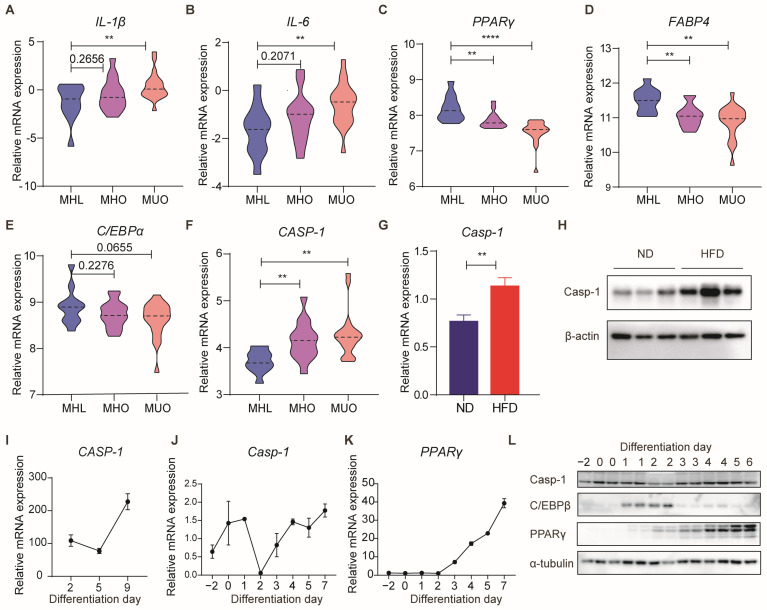
Caspase-1 expression level is correlated with obesity and adipogenesis. (**A**–**F**) The expression of *IL-1β*, *IL-6*, *FABP4*, *C/EBPα*, *PPARγ*, and *CASPASE-1* in human sub-WAT of three groups (metabolically healthy lean (MHL), metabolically healthy obese (MHO), and metabolically unhealthy obese (MUO)) (GSE152991). (**G**) qPCR analysis of *Caspase-1* in sub-WAT from HFD mice compared to respective controls (*n* = 5/group). (**H**) Western blot analysis of Caspase-1 in sub-WAT from HFD mice compared to respective controls (*n* = 5/group). (**I**) The expression of *CASP-1* in differentiating human adipose-derived stem cells (GSE237151). (**J**,**K**) qPCR analysis of *Caspase-1* and *PPARγ* in differentiating mouse C3H adipocytes. (**L**) Western blot analysis of Caspase-1 and adipogenesis-related protein levels in differentiating mouse 3T3-L1adipocytes (Original Western blot images are contained in Supplementary Materials). ** *p* < 0.01, and **** *p* < 0.0001 by Student’s *t*-test. Data presented as mean ± SEM.

During obesity, adipose tissue undergoes expansion accompanied by inflammation, concomitant with decreased adipogenesis [8,9,28,29]. As a critical inflammasome component, Caspase-1 influences the inflammatory milieu in obesity. Hence, we speculated whether Caspase-1 also plays a role in adipogenesis. According to the World Health Organization (WHO) guidelines, individuals with a BMI > 30 kg/m^2^ are classified as obese. We analyzed human data from dataset GSE152991. In GSE152991, volunteers were subsequently categorized into three groups based on BMI and metabolic indicators: metabolically healthy lean (MHL), metabolically healthy obese (MHO), and metabolically unhealthy obese (MUO). With worsening obesity and metabolic dysfunction, the expression of inflammatory factors IL-1β and IL-6 in human subcutaneous white adipose tissue (sub-WAT) escalated (Figure 1A,B), while genes related to adipogenesis were decreased (Figure 1C–E). Furthermore, the expression of *CASPASE-1* was also significantly upregulated (Figure 1F). This pattern was consistent with the substantial upregulation of Caspase-1 protein expression and mRNA levels in the sub-WAT of mice fed with a high-fat diet (HFD) for 20 weeks (Figure 1G,H). These findings suggest that Caspase-1 may influence obesity and adipogenesis in both human and mouse adipose tissue.

To delve deeper into the role of Caspase-1 in adipocyte differentiation, we analyzed data from dataset GSE237151. Interestingly, the expression of *CASPASE-1* was decreased on day 4 and increased on day 9 during the adipogenic differentiation of human adipose-derived stem cells (Figure 1I). Moreover, in a 3T3-L1-induced adipocyte differentiation model, both the RNA and protein levels of Caspase-1 exhibited consistent changes, being significantly downregulated on the second day of adipocyte differentiation and gradually increasing after that (Figure 1J–L). These observations suggest that Caspase-1 plays a crucial role in adipogenesis within adipocytes.

### 3.2. Deficiency of Caspase-1 Improves Glucose Homeostasis, Accompanied by an Increase in White Adipose Tissue, under a Normal Diet

**Figure 2:**
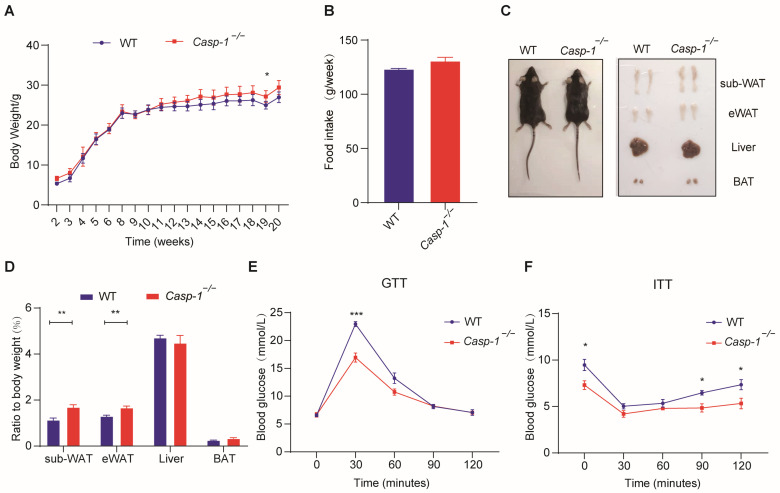
Under normal diet, Casp1^−/−^ mice exhibit increased adipose tissue mass alongside better glucose homeostasis. (**A**) Body weight of WT and *Casp-1*^−/−^ mice fed with ND (20 weeks) (*n* = 4/group). (**B**) Average food intake of WT and *Casp-1*^−/−^ mice during normal diet feeding (*n* = 4/group). (**C**) Macroscopic view of *Casp-1*^−/−^ mice and WT controls at the age of 20 weeks. (**D**) Adipose depot and liver weight of WT and *Casp-1*^−/−^ mice at the age of 20 weeks (*n* = 4). (**E**) Glucose tolerance test (GTT) performed on WT and *Casp-1*^−/−^ mice after 20 weeks of normal diet (*n* = 4). (**F**) Insulin tolerance test (ITT) performed on WT and *Casp-1*^−/−^ mice after 20 weeks of normal diet (*n* = 4) * *p* < 0.05, ** *p* < 0.01, and *** *p* < 0.001 by Student’s *t*-test. Data presented as mean ± SEM.

To investigate the impact of Caspase-1 on energy metabolism in vivo, we conducted experiments using both *Casp-1*^−/−^ and WT mice. The data showed that following 20 weeks of a regular diet, the food intake of *Casp-1*^−/−^ mice mirrored that of the WT mice (Figure 2B). However, there was a tendency for the *Casp-1*^−/−^ mice to exhibit higher body weight values (Figure 2A), albeit this disparity lacked statistical significance. Notably, the white adipose tissue in the *Casp-1*^−/−^ mice had significantly increased comparing to their WT counterparts (Figure 2C,D). This augmentation in WAT was concomitant with improved glucose tolerance and insulin sensitivity (Figure 2E,F). These compelling findings underscore that Caspase-1 deficiency under normal dietary conditions can markedly enhance glucose homeostasis in vivo, coupled with the expansion of white adipose tissue.

### 3.3. Caspase-1 Deficiency Promotes Adipogenesis In Vitro and In Vivo

**Figure 3:**
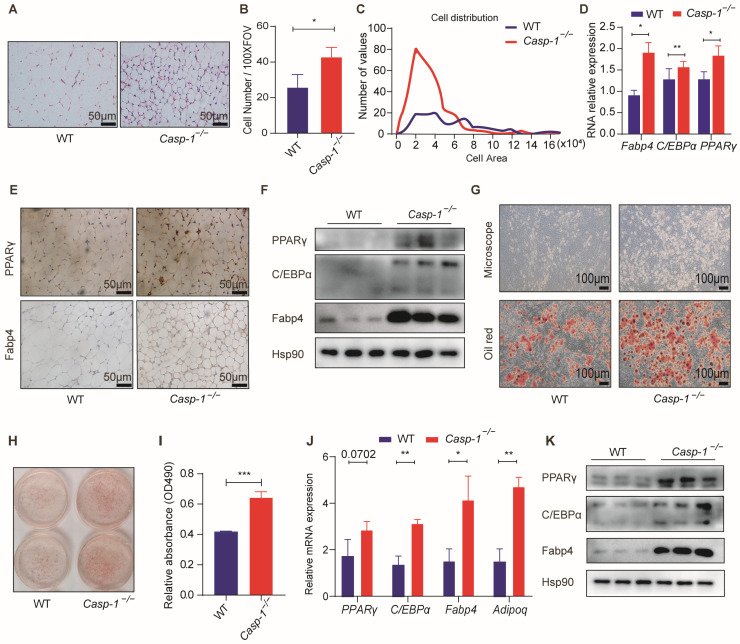
Caspase-1 deficiency promotes adipogenesis in mice sub-WAT and in primary adipocytes. (**A**) Images of H&E-stained sub-WAT from WT and *Casp-1*^−/−^ mice fed with ND. Scale bar, 20 μm. (**B**) Statistical analysis of the number of adipocytes in each field of vision. (**C**) Quantification of adipocyte size in H&E-stained sub-WAT. (**D**) qPCR analysis of the major markers of adipogenesis (*C/EBPα*, *Fabp4*, *PPARγ*) in sub-WAT from WT and *Casp-1*^−/−^ mice fed with ND (*n* = 5 mice/group). (**E**) Histological analysis of Fabp4 and PPARγ expression (IHC) in sub-WAT from WT or *Casp-1*^−/−^ mice (*n* = 3/group). Scale bar, 20 μm. (**F**) Western blot analysis of major markers of adipogenesis (C/EBPα, Fabp4, PPARγ) in sub-WAT derived from *Casp-1*^−/−^ mice compared with WT mice (Original Western blot images are contained in Supplementary Materials). (**G**,**H**) Oil Red O staining on the 6th day of treated primary sub-WAT SVF cells separated from WT and *Casp-1*^−/−^ mice. Scale bar, 100 µm. (**I**) Oil Red O-stained cells were quantified by relative absorbance at 490 nm using microplate reader (*n* = 3/group). (**J**) qPCR analysis of major markers of adipogenesis (*C/EBPα*, *Fabp4*, *PPARγ*, *Adipoq*) in differentiated primary sub-WAT SVF cells from WT and *Casp-1*^−/−^ mice (*n* = 3/group). (**K**) Western blot analysis of major markers of adipogenesis (C/EBPα, Fabp4, PPARγ) in differentiated primary sub-WAT SVF cells from WT and *Casp-1*^−/−^ mice (*n* = 3/group). * *p* < 0.05, ** *p* < 0.01, and *** *p* < 0.001 by Student’s *t*-test. Data presented as mean ± SEM.

Existing research shows that adipogenesis is a beneficial mechanism for combating obesity [9,10], a notion reinforced by our previous findings highlighting its correlation with enhanced insulin sensitivity and glucose homeostasis. To investigate the underlying rationale behind the dual effect of Caspase-1 deficiency–improved glucose homeostasis alongside increased adipose tissue size, adipose tissue analysis was performed through H&E staining and a subsequent statistical examination (Figure 3A). Remarkably, our examination showed that the adipocytes within the sub-WAT of the *Casp-1*^−/−^ mice exhibited a decrease in size compared to their WT counterparts, accompanied by a heightened adipocyte density per unit area (Figure 3B,C). Furthermore, both RNA and protein analyses unveiled a significant upregulation of markers pertinent to adipogenesis (Fabp4, C/EBPα, PPARγ) in the sub-WAT of the *Casp-1*^−/−^ mice relative to the controls (Figure 3D–F). To further confirm the role of Caspasse-1 in adipogenesis in vitro, primary preadipocytes, isolated as a stromal vascular fraction (SVF), were subjected to an adipogenic inducer cocktail. Strikingly, Oil Red O staining exhibited a more significant accumulation of lipids in the cells derived from the *Casp-1*^−/−^ mice (Figure 3G–I), coupled with a marked increase in the expression of adipogenesis-related genes in the SVF-differentiated adipocytes from the *Casp-1*^−/−^ mice (Figure 3J,K). Overall, these results suggest that the absence of Caspase-1 potentiates adipogenesis both in vitro and in vivo, thus highlighting its potential as a therapeutic target for combating obesity.

### 3.4. Deficiency of Caspase-1 Enhances Autophagy in Adipocytes

**Figure 4:**
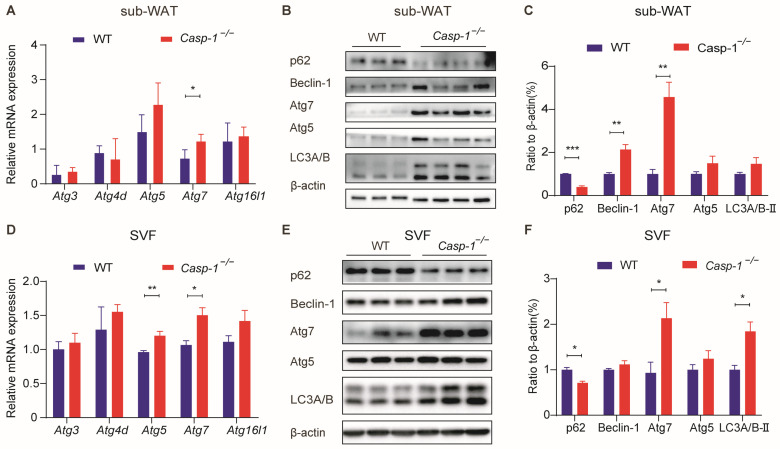
Caspase-1 deficiency promotes autophagy in mice sub-WAT and in primary adipocytes. (**A**) qPCR analysis of the major markers of autophagy in sub-WAT from WT and *Casp-1*^−/−^ mice fed with ND (*n* = 3/group). (**B**) Western blot analysis of major markers of autophagy in sub-WAT from WT and *Casp-1*^−/−^ *mice* fed with ND (*n* = 3–4/group). (**C**) Quantitation of autophagy-related protein levels in (**B**) and normalized to β-actin protein (*n* = 3/group). (**D**) qPCR analysis of major markers of autophagy in differentiated primary sub-WAT SVF cells from WT and *Casp-1*^−/−^ mice (*n* = 3/group). (**E**) Western blot analysis of major markers of autophagy in differentiated primary sub-WAT SVF cells from WT and *Casp-1*^−/−^ mice (*n* = 3/group). (**F**) Quantitation of autophagy-related protein levels in (**E**) and normalized to β-actin protein (*n* = 3/group). * *p* < 0.05, ** *p* < 0.01, and *** *p* < 0.001 by Student’s *t*-test. Data presented as mean ± SEM. (Original Western blot images are contained in Supplementary Materials).

Numerous pieces of evidence show the critical role of autophagy, a fundamental cellular process, in regulating adipogenesis [16,30,31]. Recent studies have reported the involvement of the Caspase-1-related NLRP3 inflammasome in modulating autophagy in PrP106-126-treated microglia [32], prompting our investigation into the potential role of Caspasse-1 in adipocytes. The autophagy-related protein family (Atg) is pivotal in autophagosome formation and the autophagic process [33]. Therefore, we examined the gene expression levels of the Atg family in differentiated primary adipocytes and sub-WAT. The results showed a significant increase in *Atg7* expression following Caspase-1 knockout, indicating its potential regulatory role (Figure 4A,D). To further validate the effect of Caspase-1 deficiency on autophagy, we further analyzed the key autophagy proteins via Western blot (WB). Among these proteins, P62 is a critical receptor in autophagy which can bind to autophagic substrates, and it has a negative correlation with autophagic activity. Furthermore, converting LC3B-I to LC3B-II signifies autophagic flux activation, while Beclin-1, Atg5, and Atg7 are integral to the process. The WB results showed a significant decrease in p62 expression and an increase in the expression of other autophagy proteins following Caspase-1 deficiency in both in vivo and in vitro experiments compared to the control group (Figure 4B,C,E,F). These findings strongly suggest that Caspase-1 deficiency positively regulates autophagy, highlighting its potential as a therapeutic target for modulating adipocyte function both in vivo and in vitro.

### 3.5. Deficiency of Caspase-1 Promotes Adipogenesis by Enhancing Autophagy

**Figure 5:**
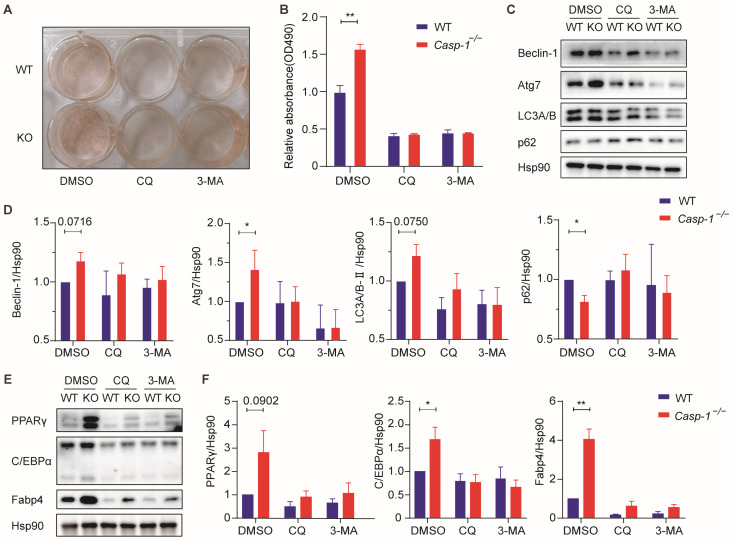
Treatment with either CQ or 3-MA in primary adipocytes attenuated the enhancement of adipogenesis due to Caspase-1 deficiency. (**A**) Images of Oil Red O staining in the presence of 3-MA or CQ. 3MA, 3-methyladenine. CQ, chloroquine. (**B**) Oil Red O-stained cells were quantified by relative absorbance at 490 nm using microplate reader (*n* = 3/group). (**C**) Western blot analysis of autophagy-related proteins from differentiated primary sub-WAT SVF cells from WT and *Casp-1*^−/−^ mice in the presence of 3-MA or CQ (*n* = 3/group). (**D**) Quantitation of autophagy-related protein levels in (**C**) and normalized to Hsp90 protein (*n* = 3/group). (**E**) Western blot analysis of major markers of adipogenesis (C/EBPα, Fabp4, PPARγ) from differentiated primary sub-WAT SVF cells from WT and *Casp-1*^−/−^ mice in the presence of 3-MA or CQ (*n* = 3/group). (**F**) Quantitation of C/EBPα, PPARγ and Fabp4 protein levels in (**E**) and normalized to β-actin protein (*n* = 3/group). * *p* < 0.05 and ** *p* < 0.01 by Student’s *t*-test. Data presented as mean ± SEM. (Original Western blot images are contained in Supplementary Materials).

To explore whether Caspasse-1 modulates adipogenesis via autophagy, the SVF derived from *Casp-1*^−/−^ mice and WT mice were subjected to adipogenic differentiation with or without the autophagy inhibitors 3-methyladenine (3-MA) and chloroquine (CQ) [34,35]. The Oil Red O staining depicted that treatment with 3-MA or CQ attenuated Caspase-1 deficiency’s promotion of adipogenesis (Figure 5A,B). Consistent with those findings, the expression of autophagy marker genes mirrored this trend (Figure 5C,D). Notably, the upregulation of adipogenesis-related genes such as C/EBPα, PPARγ, and Fabp4 observed in the adipocytes from the *Casp-1*^−/−^ mice were significantly reversed by 3-MA or CQ treatment (Figure 5E,F). These data collectively reveal the pivotal role of Caspase-1 deficiency in enhancing adipogenesis by promoting autophagy. To explore whether Caspasse-1 regulates adipogenesis via autophagy, the SVF cells isolated from the *Casp-1*^−/−^ mice and WT mice were treated with or without autophagy inhibitor 3-methyladenine (3-MA) and chloroquine (CQ) during adipogenic differentiation [29,30]. The Oil Red O staining showed that 3-MA or CQ treatment attenuated the promotion of adipogenesis under Caspase-1 deficiency (Figure 5A,B). Consistently, as well as autophagy marker genes (Figure 5C,D), the expression of C/EBPα, PPARγ and Fabp4 were remarkably upregulated in adipocytes from *Casp-1*^−/−^ mice but reversed by 3-MA or CQ treatment (Figure 5E,F). Collectively, these data reveal that Caspase-1 deficiency enhances adipogenesis by promoting autophagy.

### 3.6. Caspase-1 Deficiency Enhances Autophagy and Adipogenesis through Atg7

**Figure 6:**
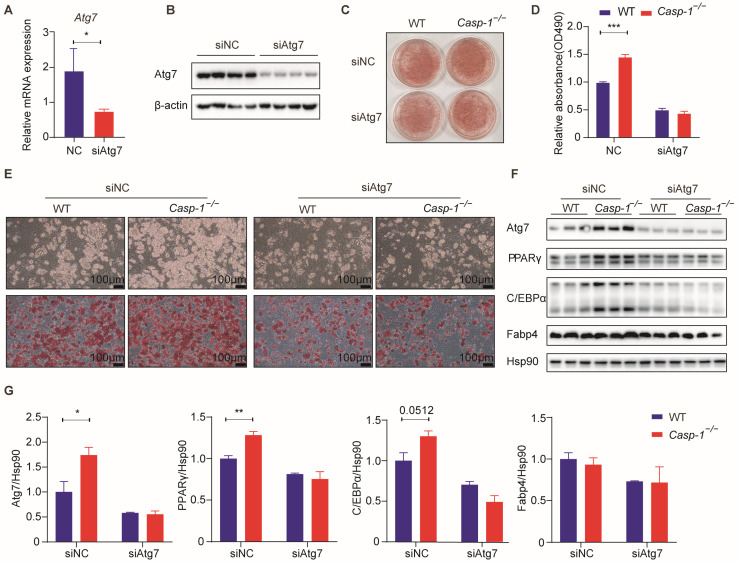
The improvement in adipogenesis resulting from Caspase-1 deletion is suppressed upon Atg7 knockdown in the primary adipocytes of mice. (**A**) qPCR analysis of *Atg7* verified the effect of siATG7 in 293T cells (*n* = 3/group). (**B**) Western blot analysis of Atg7 verified the effect of siATG7 in 293T cell (*n* = 3/group). (**C**–**E**) The representative Oil Red O staining images and relative Oil Red O OD values showed the different lipid accumulations in primary sub-WAT SVF cells treated with si-NC and si-Atg7 after the induction of adipogenic differentiation. Scale bar = 100 µm. (**F**) Western blot analysis of autophagy-related proteins in primary sub-WAT SVF cells treated with si-NC and si-Atg7 after the induction of adipogenic differentiation. (*n* = 3/group). (**G**) Quantitation of autophagy-related protein levels in (**F**) and normalized to Hsp90 protein (*n* = 3/group). * *p* < 0.05, ** *p* < 0.01, and *** *p* < 0.001 by Student’s *t*-test. Data presented as mean ± SEM. (Original Western blot images are contained in Supplementary Materials).

Various studies have shown Atg7, a pivotal protein in the autophagic process, to regulate adipogenesis. Our experimental results corroborated this, revealing a significant increase in Atg7 expression at both gene and protein levels in the absence of Caspase-1, evident in both in vivo and in vitro experiments (Figure 4). To elucidate whether Atg7 is indispensable for the Caspase-1 deficiency-induced promotion of adipogenesis, we conducted a rescue experiment by knocking down Atg7 in SVF cells derived from *Casp-1*^−/−^ mice. Small interfering RNA (siRNA) effectively attenuated the expression of Atg7 (Figure 6A,B,F). Furthermore, the depletion of Atg7 successfully reversed the heightened adipogenesis and lipid accumulation observed in the adipocytes from the *Casp-1*^−/−^ mice (Figure 6C–E). Consistently, the upregulated expression of the adipogenic markers C/EBPα, PPARγ and Fabp4, triggered by Caspase-1 deficiency, was also ameliorated upon Atg7 knockdown (Figure 6F,G). These findings demonstrate the critical role of Atg7 in mediating the promotion of adipogenesis induced by Caspase-1 deficiency.

### 3.7. Caspase-1 Deficiency Ameliorates High-Fat Diet-Induced Obesity and Glucose Intolerance

**Figure 7:**
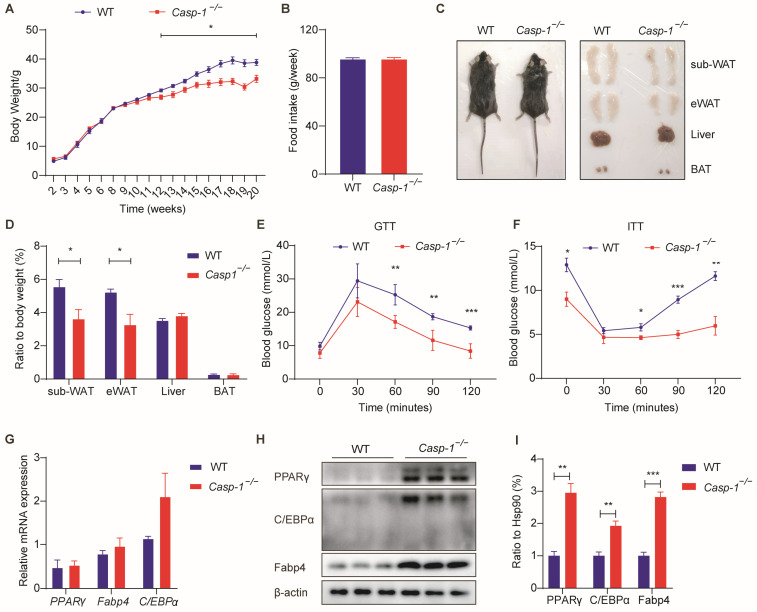
Caspase-1 deficiency ameliorates high-fat diet-induced obesity and glucose intolerance. (**A**) Body weight of WT and *Casp-1*^−/−^ mice fed with HFD (12 weeks) (*n* = 4/group). (**B**) Average food intake of WT and *Casp-1*^−/−^ mice during high-fat diet feeding (*n* = 4/group). (**C**) Macroscopic view of *Casp-1*^−/−^ mice and WT controls at the age of 20 weeks after 12 weeks of high-fat diet feeding. (**D**) Adipose depot and liver weight of WT and *Casp-1*^−/−^ mice after 12 weeks of high-fat diet feeding. (**E**) Glucose tolerance test (GTT) performed on WT and *Casp-1*^−/−^ mice at the age of 20 weeks after 12 weeks of high-fat diet feeding (*n* = 4/group). (**F**) Insulin tolerance test (ITT) performed on WT and *Casp-1*^−/−^ mice at the age of 20 weeks after 12 weeks of high-fat diet feeding (*n* = 4/group). (**G**) qPCR analysis of major markers of adipogenesis (*C/EBPα*, *Fabp4*, *PPARγ*) in sub-WAT from WT and *Casp-1*^−/−^ mice fed with HFD (*n* = 4/group). (**H**) Western blot analysis of major markers of adipogenesis (C/EBPα, Fabp4, PPARγ) in sub-WAT derived from *Casp-1*^−/−^ mice compared with WT (*n* = 4/group). (**I**) Quantitation of C/EBPα, PPARγ and Fabp4 protein levels in (**H**) and normalized to α-tubulin protein (*n* = 4/group). * *p* < 0.05, ** *p* < 0.01, and *** *p* < 0.001 by Student’s *t*-test. Data presented as mean ± SEM. (Original Western blot images are contained in Supplementary Materials).

Previous experiments demonstrated that Caspase-1 deficiency promotes adipogenesis through Atg7-mediated autophagy; we established an animal model by subjecting mice to a high-fat diet from week 8 until week 20 to investigate the impact of Caspase-1 deficiency under obesity conditions in vivo. Strikingly, our results showed that despite comparable food intake, the weight of the *Casp-1*^−/−^ mice exhibited a significant reduction compared to the control mice as early as the twelfth week (Figure 7A,B). Moreover, overall volume and adipose tissue volume were notably diminished in the *Casp-1*^−/−^ mice (Figure 7C), and a decreased subcutaneous and visceral adipose tissue weight to body weight ratio was also found (Figure 7D). Additionally, glucose and insulin tolerance tests showed better glucose tolerance and insulin sensitivity in the *Casp-1*^−/−^ mice compared to the WT mice (Figure 7E,F). Concurrently, the protein expression analysis revealed elevated levels of the adipogenic markers PPARγ and Fabp4 in the sub-WAT of the *Casp-1*^−/−^ mice (Figure 7G–I). These data indicate that, under a high-fat diet, Caspase-1 deficiency can promote adipogenesis and confer resistance against high-fat diet-induced obesity and glucose intolerance.

## 4. Discussion

Studies have shown that adipogenesis can observably mitigate the detrimental metabolic effects associated with obesity, but adipocyte hypertrophy exacerbates metabolic disorders [8,9,10]. In the advanced stages of obesity, adipogenesis becomes markedly inhibited due to adipocyte hypertrophy. Thus, promoting adipogenesis emerges as a promising strategy for combating obesity. Furthermore, the infiltration of immune cells during obesity instigates chronic inflammation within adipose tissue, triggering the secretion of numerous inflammatory factors [28,29,36]. This inflammatory milieu not only inhibits adipogenesis but also disrupts the internal environment. For example, the inflammatory factor TNF-α promotes obesity and insulin resistance in mice and inhibits adipocyte differentiation primarily by activating TNFR1 [37,38]. Similarly, prolonged exposure to IL-6 reduces adipogenesis and glucose transport in 3T3-F442A and 3T3-L1 cells [39]. Moreover, IL-15, despite its proinflammatory nature, mediates the calcium-dependent inhibition of adipocyte differentiation in 3T3-L1 cells by upregulating α-calcineurin [40,41,42].

Previous studies have highlighted that Caspase-1, a critical protease constituting the inflammasome, for its ability to generate active IL-1β and IL-18 [43,44]. IL-1β, a renowned inhibitor of adipogenesis, exhibits upregulation in the adipose tissue of obese mice and impedes adipocyte differentiation by binding to type 1 IL-1R and activating intracellular signaling pathways, notably the NF-kB pathway [45,46,47]. Conversely, Caspase-1 deficiency has been associated with promoting adipogenesis [27]. IL-18, another important inflammatory factor produced by Caspase-1, is increased in obese individuals, and *IL-18*^−/−^ mice exhibit obesity and insulin resistance [48,49]. Nevertheless, its effects on adipogenesis and lipid metabolism remain to be further studied. However, existing studies predominantly attribute the adverse effects of adipogenesis to alterations in adipose tissue inflammation. In this study, we validated the correlation of Caspase-1 with obesity and adipogenesis in human and mouse tissues and cells (Figure 1). Under normal dietary conditions, when mice were in a state of minimal or low inflammation, the weight values of the WT mice and *Casp-1*^−/−^ mice were similar (Figure 2). Nevertheless, the *Casp-1*^−/−^ mice showed increased adipose tissue mass, while H&E staining revealed a decrease in the size of subcutaneous adipocytes, suggesting a higher adipocyte count in the *Casp-1*^−/−^ mice compared to the WT mice. Furthermore, the expression levels of the adipogenic markers C/EBPα, PPARγ, and Fabp4 were significantly upregulated in the sub-WAT of the *Casp-1*^−/−^ mice (Figure 3). Given that in vivo experiments may not entirely exclude the role of macrophages and inflammatory factors, the primary adipocyte differentiation experiments were conducted to verify the role of Caspase-1 in vitro. Strikingly consistent with the data in mice, the absence of Caspase-1 in adipocytes also promotes the differentiation process (Figure 3). These results suggested that Caspase-1 deficiency may promote adipogenesis independent of inflammation.

As a proteolytic enzyme, Caspase-1 has a broad range of substrate specificity that extends far beyond inflammation [50]. Recent findings have unveiled that Caspase-1 substrates are associated with cell death, cytoskeleton dynamics, and metabolism, suggesting its inflammasome-independent functions [50,51]. Notably, Caspase-1 has been implicated in autophagy regulation. Majid et al. highlighted Caspase-1′s ability to cleave TRIF, a TLR adapter, suppressing autophagy during Pseudomonas aeruginosa infection [32,52]. Conversely, Caspase-1 activation has been shown to safeguard hepatocytes by upregulating the Beclin-1 protein and promoting mitochondrial autophagy [53]. Moreover, some studies have reported that autophagy is involved in adipogenesis [30,31]. Guo et al. reported that activated autophagy in 3T3-L1 cells promotes adipogenic differentiation by decreasing negative adipogenic regulatory factors dependent on autophagy degradation [33]. Additionally, chloroquine treatment inhibits adipogenesis in mouse embryonic fibroblasts (MEFs) while promoting autophagy induction during adipocyte differentiation [18,54]. Our investigation noted the upregulation of autophagy-related markers in *Casp-1*^−/−^ mice (Figure 4). Notably, the enhancement of adipogenesis was reversed by inhibiting autophagy with chloroquine or 3-MA treatment (Figure 5). These results indicated that Caspase-1 deficiency promoted adipocyte differentiation by enhancing autophagy. Of particular interest, Atg7, a crucial member of the Atg family responsible for extending autophagic vacuole membranes, facilitating the coupling of Atg5 and Atg12, as indicated by previous studies [33,55]. A study has shown that under similar dietary conditions, the conditional knockout of Atg7 in Fabp4+ cells results in mice exhibiting smaller and leaner phenotypes than control mice, with a remarkable 30% reduction in total fat content, indicating that the efficiency of adipogenesis is significantly reduced after the adipocyte-specific knockout of Atg7 [56]. In our experiments, we observed a substantial increase in Atg7 expression both in subcutaneous adipose tissue from *Casp-1*^−/−^ mice and in adipocytes derived from *Casp-1*^−/−^ mice, hinting the potential of Atg7 as a potential key factor influencing autophagy and adipogenesis in our model (Figure 4). Furthermore, knocking down Atg7 attenuated the adipogenesis promotion caused by Caspase-1 deficiency (Figure 6). This result needs further exploration to determine whether the increase in the expression of Atg7 due to Caspase-1 deficiency is a result of the reduced cleavage of Atg7 protein as a substrate of Caspase-1 or if other currently unknown mechanisms are at play. Thus, the specific mechanism by which Atg7 regulates adipocyte differentiation warrants deeper investigation.

The existing reports regarding the role of Caspase-1 in obesity present conflicting findings. One study found that compared to WT mice, mice lacking Caspase-1 exhibited increased insulin sensitivity, attributed to reduced IL-1β secretion [27]. In addition, treating obese mice with a Caspase-1 inhibitor significantly improved insulin sensitivity. VanDiepen JA et al. found that in fasting animals fed with lipids, lipid absorption in the intestine decreased and lipid excretion increased, resulting in reduced adipose tissue and plasma TG levels in *Casp-1*^−/−^ mice compared to WT mice [25]. However, another study has reported opposite results, suggesting that Caspase-1-deficient mice were more susceptible to high-fat diet-induced obesity and increased inflammation, primarily through the CCL2/C-C chemokine receptor 2 (CCR2) axis in adipose tissue. The discrepancies in these findings may stem from variations in experimental methods or potential correlations with microbiota. Nevertheless, these studies indicate an intimate relationship between Caspase-1, lipid metabolism, and obesity. In our study, we observed that the absence of Caspase-1 ameliorated high-fat diet-induced obesity and improved glucose tolerance. Although in vivo experiments cannot completely rule out the role of Caspase-1 deficiency in macrophages and inflammation, the enhancement of adipogenesis likely contributes to the resistance against high-fat diet-induced obesity and glucose intolerance. Further investigation involving adipocyte-conditional knockout mice is warranted to delve deeper into these mechanisms.

Our findings highlight that Caspase-1 deficiency exerts a dual effect on adipogenesis by promoting autophagy through Atg7. Beyond its established role in upregulating the expression of immune factors to promote inflammation, Caspase-1 also directly impedes adipogenesis in obese subjects, exacerbating metabolic dysregulation. Therefore, our study underscores the multifaceted impact of Caspase-1 on obesity and body metabolism, encompassing both inflammatory processes and adipogenesis. These insights provide a novel theoretical foundation for preventing and managing obesity and metabolic syndrome.

## 5. Conclusions

In summary, our study reveals that the absence of Caspase-1 can promote adipogenesis in subcutaneous adipose tissue independently from inflammation, thereby aiding in maintaining metabolic balance. We have demonstrated that Caspase-1 deficiency promotes adipogenesis through Atg7-mediated autophagy, both in vivo and in vitro. Consequently, our findings unveil a novel mechanism through which Caspase-1 influences adipogenesis and offer fresh insights into its role in controlling and modulating lipid metabolism.

## Data Availability

The data supporting the findings of this study are available from the GEO datasets at the following links: https://www.ncbi.nlm.nih.gov/geo/query/acc.cgi?acc=GSE152991 and https://www.ncbi.nlm.nih.gov/geo/query/acc.cgi?acc=GSE237151. Additionally, all other relevant data are included in the paper.

## Supplementary Materials

The following supporting information can be downloaded at: https://www.mdpi.com/article/10.3390/biom14040501/s1., Table S1:Primers used in this research; Figure S1: Original Western blot images in Figure 1H; Figure S2: Original Western blot images in Figure 1L; Figure S3: Original Western blot images in Figure 3F; Figure S4: Original Western blot images in Figure 1K; Figure S5: Original Western blot images in Figure 4B; Figure S6: Original Western blot images in Figure 4E; Figure S7: Original Western blot images in Figure 5C; Figure S8: Original Western blot images in Figure 5E; Figure S9: Original Western blot images in Figure 6B; Figure S10: Original Western blot images in Figure 6F; Figure S11: Original Western blot images in Figure 7H.
