# Supplementary material for: Caspase-1 Deficiency Modulates Adipogenesis through Atg7-Mediated Autophagy: An Inflammatory-Independent Mechanism
Source: Biomolecules. 2024 Apr 20;14(4):501. doi: 10.3390/biom14040501 (PMC11048440; doi:10.3390/biom14040501)

Supplementary Table S1 Primers used in this research.

Primer Name	Sequence(5’ to 3’)
Caspase-1 F	ATACAACCACTCGTACACGTCTTG
Caspase-1 R	CAGATCCTCCAGCAGCAACTTC
18S F	CGCCGCTAGAGGTGAAATTCT
18S R	CATTCTTGGCAAATGCTTTCG
C/EBPα F	CAAGAACAGCAACGAGTACCG
C/EBPα R	GTCACTGGTCAACTCCAGCAC
FABP4 F	ACACCGAGATTTCCTTCAAACTG
Fabp4 R	CCATCTAGGGTTATGATGCTCTTCA
PPARγ F	GTGCCAGTTTCGATCCGTAGA
PPARγ R	GGCCAGCATCGTGTAGATGA
Adipoq F	TGGAGAAGCCGCTTATGTGTATCG
Adipoq R	TGCTGCCGTCATAATGATTCTGTTG
Atg3 F	GGCAGTTTTTGACTCCCCTG
Atg3 R	TTCCCCTGTAGCCCTCTTCT
Atg4d F	CCCCGGCATTCACTGTACTT
Atg4d R	TGGCAAAGGCCATCTCCAG
Atg5 F	TGTGCTTCGAGATGTGTGGTT
Atg5 R	GTCAAATAGCTGACTCTTGGCAA
Atg7 F	GCTGCTGAGATCTGGGACAT
Atg7 R	GAGATGTGGAGATCAGGACCAG
Atg16l1 F	TCCGAATCTCCCCTTTTGGGA
Atg16l1 R	CTGCACTGCGTTGACCTCTC

Supplementary Figure S1. Original Western blot images in Figure 1H.

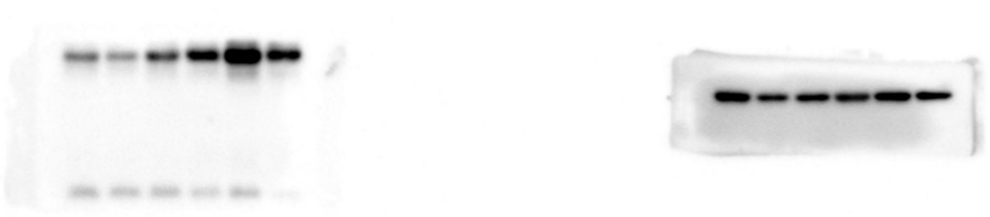

Supplementary Figure S2. Original Western blot images in Figure 1L.

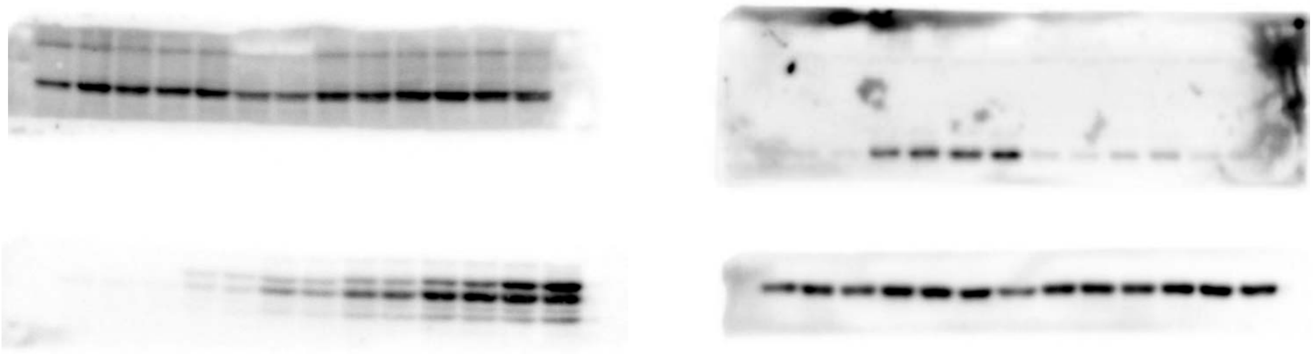

Supplementary Figure S3. Original Western blot images in Figure 3F.

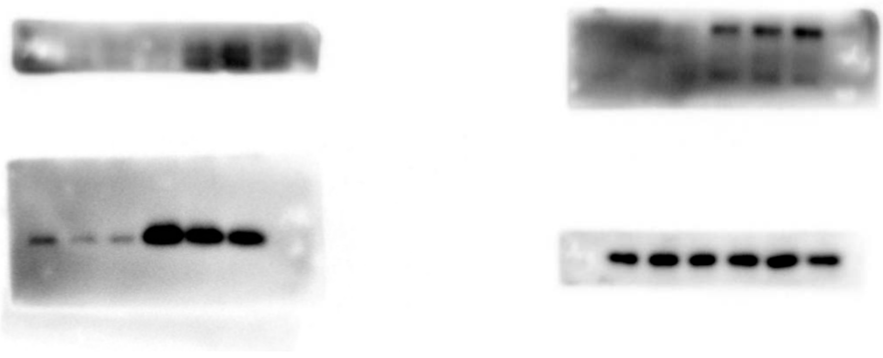

Supplementary Figure S4. Original Western blot images in Figure 3K.

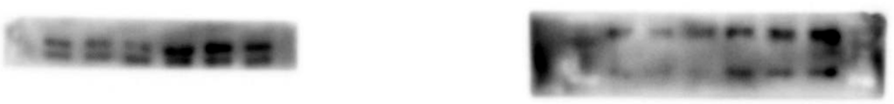

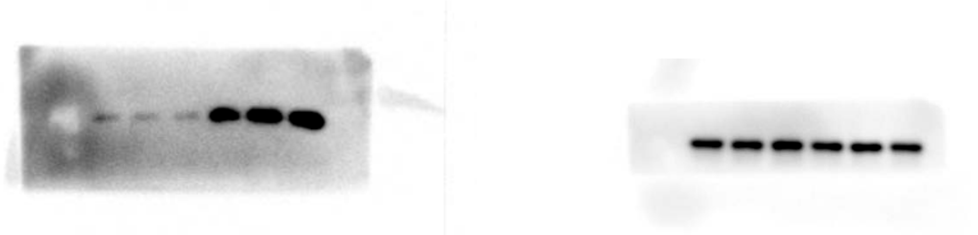

Supplementary Figure S5. Original Western blot images in Figure 4B.

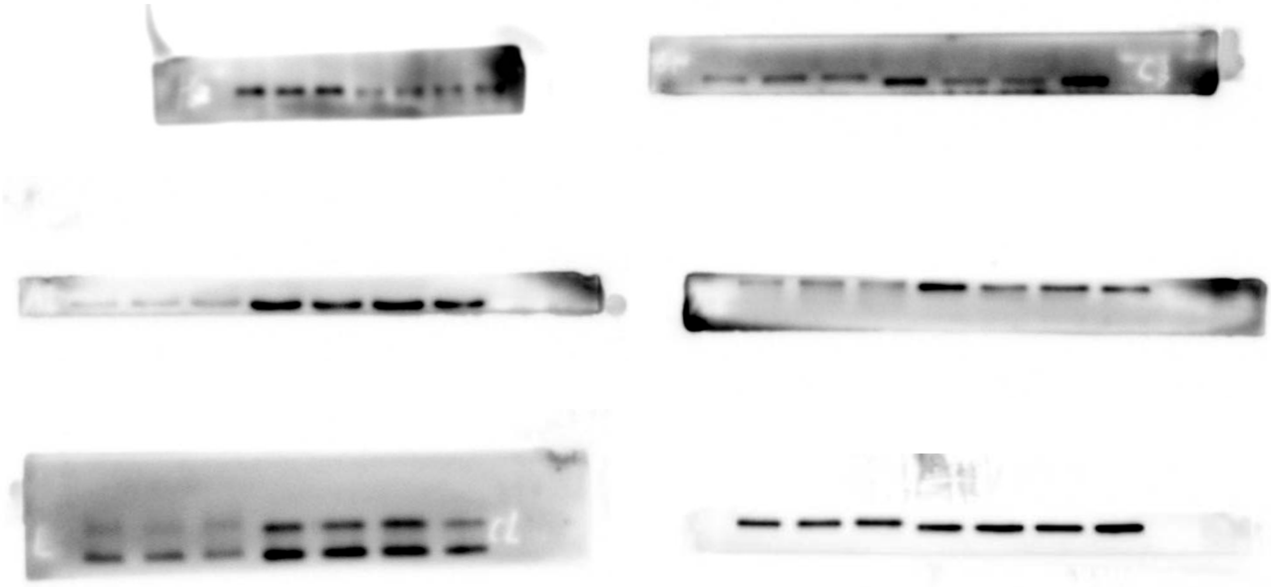

Supplementary Figure S6. Original Western blot images in Figure 4E.

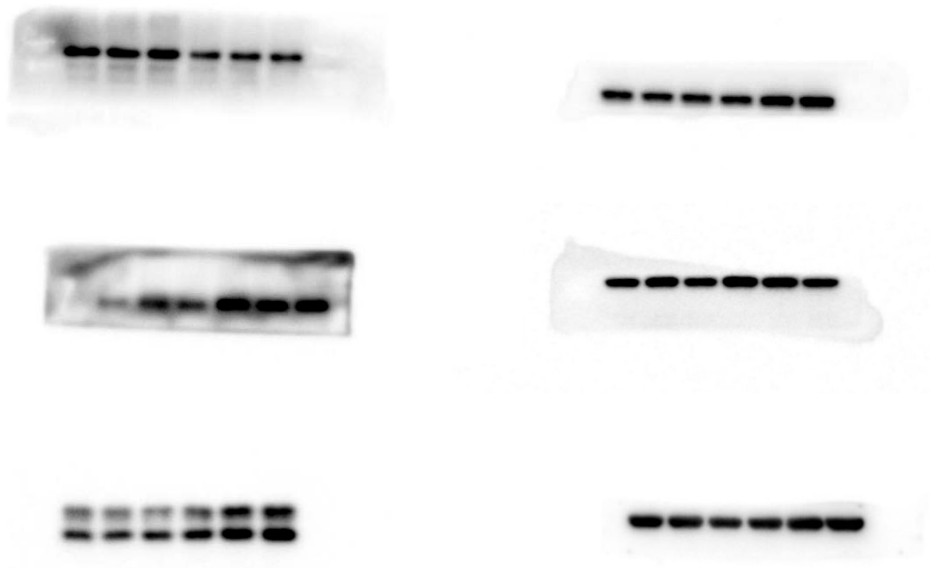

Supplementary Figure S7. Original Western blot images in Figure 5C.

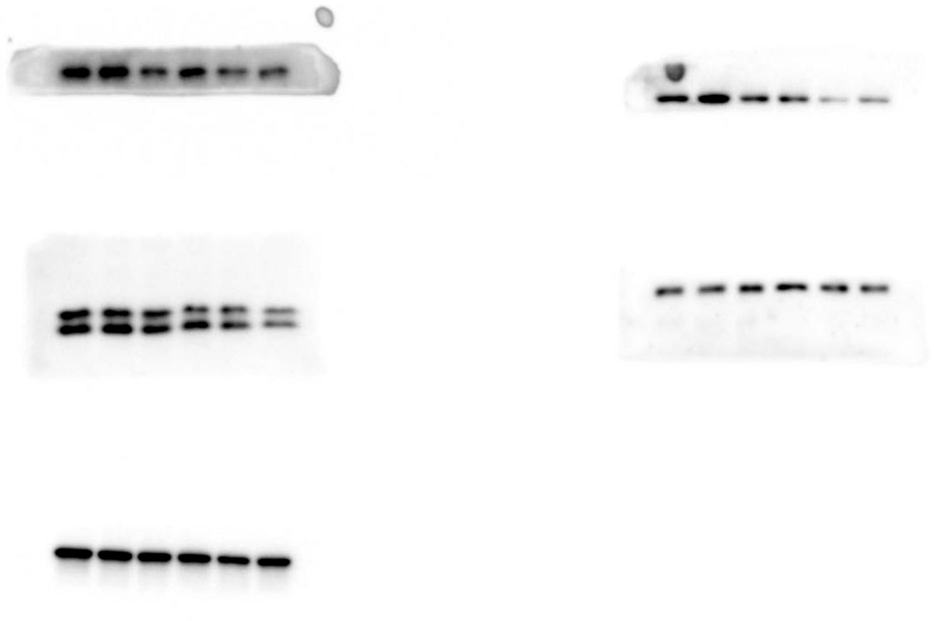

Supplementary Figure S8. Original Western blot images in Figure 5E.

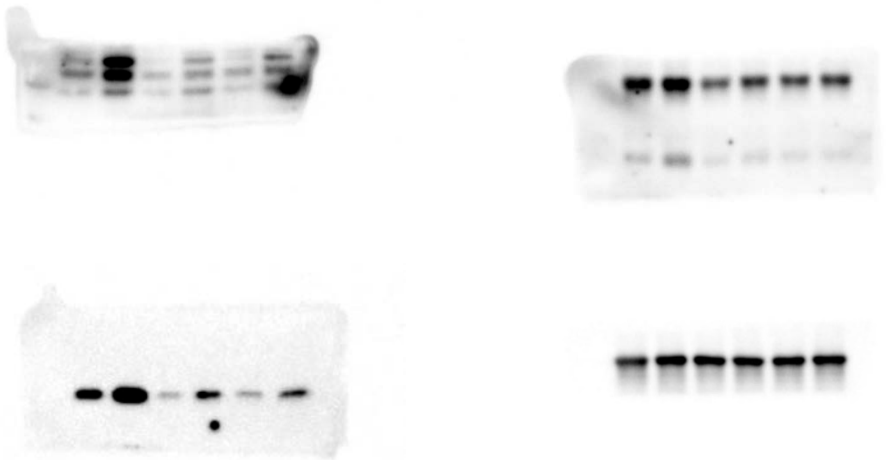

Supplementary Figure S9. Original Western blot images in Figure 6B.

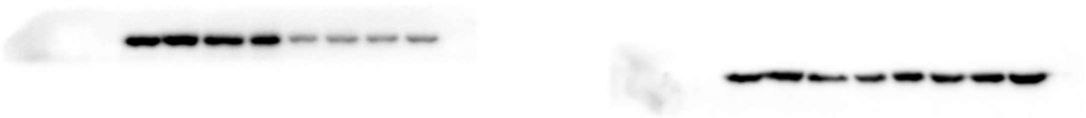

Supplementary Figure S10. Original Western blot images in Figure 6F.

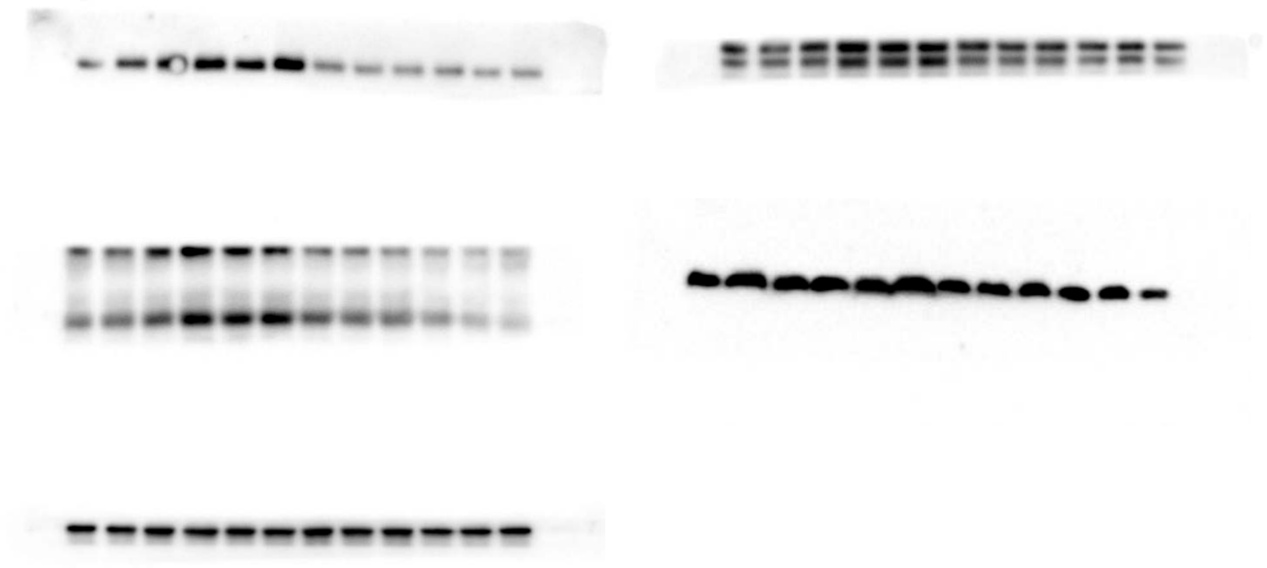

Supplementary Figure S11. Original Western blot images in Figure 7H.

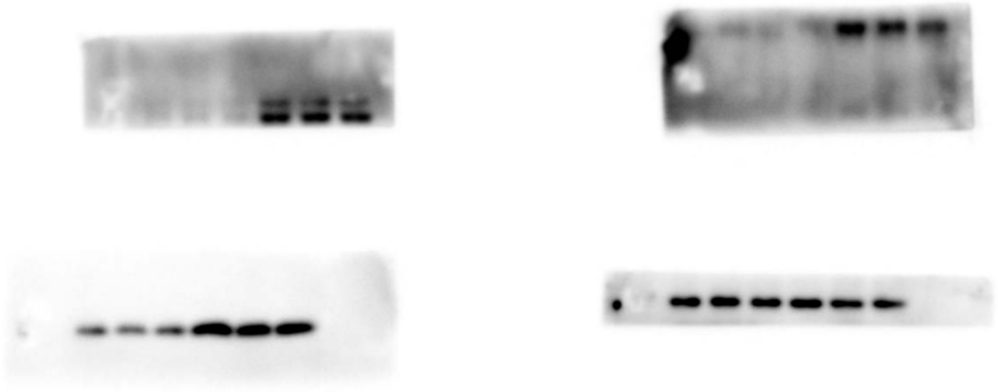

Supplement: Supplementary file 1 [file biomolecules-14-00501-s001.zip › biomolecules-2970646-supplementary.pdf]
